# Barriers and Facilitators to Physical Activity in People With Young‐Onset (Aged 18‐40 Years) Type 2 Diabetes: A Qualitative Study

**DOI:** 10.1111/jocn.17691

**Published:** 2025-02-18

**Authors:** Xiaoyan Zhao, Maria Duaso, Haya Abu Ghazaleh, Xiaodi Guo, Angus Forbes

**Affiliations:** ^1^ Care for Long Term Conditions Division, Florence Nightingale Faculty of Nursing, Midwifery & Palliative Care King's College London London UK; ^2^ The Third Affiliated Hospital Sun Yat‐Sen University Guangzhou China

## Abstract

**Aim:**

To explore the barriers and facilitators to physical activity engagement among people with young‐onset type 2 diabetes.

**Design:**

A qualitative research design using individual semi‐structured interviews.

**Methods:**

A purposive sampling technique was used to recruit individuals with young‐onset type 2 diabetes through social media, based on: age, gender, diabetes duration, diabetes complication and physical activity level. Interviews were audio recorded, transcribed verbatim and analysed using Framework analysis integrating the Capability, Opportunity, Motivation and Behaviour model.

**Results:**

Twenty‐three individuals with type 2 diabetes (median age 29 years; 13 women; median diabetes duration 1 year) were interviewed. Nineteen subthemes were identified across all domains of the Capability, Opportunity, Motivation and Behaviour model. The most common domains and the related subthemes were psychological capability (physical activity knowledge, self‐monitoring); social opportunity (stigma, family commitments, guidance from professionals, interactive physical activity, emotional support); and reflective motivation (perceived physical impact of physical activity, perceived mental impact of physical activity, social role & responsibility, perceived self‐efficacy). Interactions were also observed between the different domains of the Capability, Opportunity, Motivation and Behaviour model.

**Conclusion:**

This study revealed in‐depth and novel information on the barriers and facilitators to physical activity in people with young‐onset type 2 diabetes. Future interventions would require multimodal approaches to enhance physical activity motivation in this population by addressing these underpinning psychological and social barriers.

**Implications for the Profession and Patient Care:**

This study highlighted the need for a multimodal strategy that addresses psychological capability, social opportunity and reflective motivation for increasing physical activity in people with young‐onset type 2 diabetes.

**Reporting Method:**

This study was reported using the Consolidated Criteria for Reporting Qualitative Research checklist.

**Patient or Public Contribution:**

An advisory group including six individuals with young‐onset type 2 diabetes contributed to the design of the interview topic guide.

**Summary**

- What does this paper contribute to the wider global community?
  ○ This study makes an important contribution to knowledge on barriers and facilitators to physical activity in people with young‐onset type 2 diabetes.
  ○ The findings illustrate that to support physical activity, it is necessary to attend to the social context in which behaviours occur as well as provide motivational support.
  ○ The study emphasises the need to develop multimodal interventions incorporating psychological, social and motivational components to enhance physical activity in people with young‐onset type 2 diabetes.

## 1 Introduction and Background

Diabetes presents a major public health challenge. The prevalence of diabetes continues to increase, current estimates suggest that one in ten people have diabetes globally, the majority of whom have type 2 diabetes (International Diabetes Federation 2021). The highest prevalence of type 2 diabetes is in China, this has been driven by rapid economic development and urbanisation, leading to increasingly sedentary lifestyles and rising obesity levels (Li et al. 2020; Zhao et al. 2021). A particular concern is the rapidly increasing incidence of type 2 diabetes in younger adults (18–40 years), a recent national survey in China reported that the prevalence of diabetes was 2.0% in people aged 18–29 years and 6.3% in those aged 30–39 years (Li et al. 2020). Young‐onset (18–40 years) type 2 is associated with increased risk of diabetes complications compared to those diagnosed in older age. A growing body of evidence shows that people with young‐onset type 2 diabetes have a faster deterioration of β‐cell function, higher risk of cardiovascular disease and all‐cause mortality and lower life expectancy (Kaptoge et al. 2023; Magliano et al. 2020; Zhao et al. 2021). Young‐onset diabetes also bring significant economic pressures both in terms of productivity loss and managing diabetes complications (Liu et al. 2023). Being diagnosed with type 2 diabetes at younger age (18–40 years) is also associated with psychological morbidity and reduced quality of life, with lower self‐compassion, and higher levels of depression, diabetes distress and stigma (Barker et al. 2023; Lascar et al. 2018).

Physical activity is defined as all body movement that increases energy use (Kanaley et al. 2022). It is an important component of diabetes self‐management and increased or sustained physical activity has been shown to improve physical and psychological health in people with type 2 diabetes (Kanaley et al. 2022; Magalhães et al. 2019; Motiani et al. 2020). Data suggests that 30% increment in physical activity would reduce 5.3% deaths due to type 2 diabetes, and a 10% relative increment in physical activity would reduce stroke deaths by 19.9% (Medina et al. 2020). However, current global estimates reported that > 25% of adults are insufficiently physical active, and the data suggest that activity levels continue to decline (Guthold et al. 2018; Ng and Popkin 2012). Furthermore, physical activity levels in people with diabetes have been shown to be 40% lower than those without diabetes (Zhao et al. 2020). As an emerging population, there is currently lack of effective interventions to support the young‐onset type 2 diabetes population. Current interventions are generic and do not address some of unique challenges that can impact on engagement in physical activities in younger population, such as work and childcare commitments (Song and Frier 2022). Hence, interventions tailored to the different factors impact younger adults with type 2 diabetes are needed.

Previous studies have sought to explore the factors that impact on physical activity in people with type 2 diabetes; however, most of these studies were not exclusively focussed on younger adults with type 2 diabetes (Bethancourt et al. 2014; Guthold et al. 2018; Maula et al. 2019; Peng et al. 2023; Wong et al. 2023). This paper presents the findings of study that aimed to explicate the barriers and facilitators to physical activity experienced by people with young‐onset type 2 diabetes.

## 2 Methods

### 2.1 Design

This study employed a descriptive qualitative research design and utilised individual semi‐structured interviews to collect data. The data were analysed using Framework analysis, the Capability, Opportunity, Motivation and Behaviour (COM‐B) model was used to provide a theoretical framework for the analysis (Michie et al. 2011). The COM‐B has been widely used as a framework for behavioural interventions to promote physical activity (Haley et al. 2023). The Consolidated Criteria for Reporting Qualitative Research (COREQ) checklist was used to report findings in this qualitative study (Tong et al. 2007).

### 2.2 Study Aim

The aims of the study were two‐fold:

1. To explore the experience and perceptions toward physical activity in people with young‐onset type 2 diabetes.
2. To identify potential barriers and facilitators to physical activity.

### 2.3 Sample and Recruitment

Purposive sampling (Patton 2002) was used to recruit individuals with young‐onset type 2 diabetes, ensuring a balanced representation of age (around half aged 18–29 years and half aged 30–40 years) and gender (approximately equal proportions of males and females). Additionally, the sample included a diverse range of diabetes durations, complications and physical activity levels. Potential participants were recruited using recruitment advertisements (Appendix S1) in online diabetes groups on WeChat (which is the most used social media platform in China) from July 2023 to September 2023. Eligible participants are those diagnosed with type 2 diabetes for at least three months, aged 18 to 40 years old, of Chinese origin, residing in mainland China, and fluent in Mandarin. Exclusion criteria included as follows: lack capacity to consent. Participants responded to advertisements by contacting the researcher, and their eligibility was assessed. If they were eligible, they were given the participant information sheet and a consent form to complete. Participants were given a minimum of 24 h to review the information and provide informed consent. If they agreed to participate and provided consent form, an in‐depth, semi‐structured one‐to‐one interview was arranged. Participants were informed through the recruitment advertisement that a payment of ¥50 RMB ($7) was available for participating in the interview. Sample size was determined using the concept of data saturation (Moser and Korstjens 2018); this is the point where no new analytical information was obtained. This occurred at the twentieth interview, after which three more individuals were interviewed to increase saturation certainty.

### 2.4 Data Collection

Individual semi‐structured interviews were carried out online (Microsoft Teams or Zoom) in Mandarin and audio recorded. Pilot interviews (*n* = 2) took place to assess and refine the interview topic guide (Appendix S1). The questions in the interview topic guide were informed by the COM‐B model to explore participants' capability, opportunity and motivation for physical activity. Interviews were conducted by X.Z. (a female PhD student who is a native Mandarin speaker) and lasted between 30 to 80 min with no repeat interviews. X.Z. has attended courses and received training in advanced qualitative research methods. No individuals aside from the participants and researchers were present during the interviews. Field notes were taken throughout the interviews. No relationship was established between the interviewer and participants before the study commencement. Demographic and clinical data were also collected during the interview schedule. Participants were given ¥50 RMB ($7) as a gesture of respect and appreciation for their time and effort in contributing to this research.

### 2.5 Data Analysis

All interviews including pilot interviews were transcribed verbatim by X.Z. and uploaded to NVivo 12 software for data management. Two transcripts were initially coded by three authors (X.Z., M.D., H.A.G.) independently, and disagreements were resolved through discussion with a fourth reviewer (A.F.). All transcripts were reviewed for accuracy and translated into English by the researchers who were bilingual (X.Z., X.G.) to enable analysis by English‐speaking co‐authors. All themes and codes were discussed within the team. Framework analysis (Gale et al. 2013) which incorporated both inductive and deductive reasoning across its seven stages: (1) verbatim transcription in Mandarin for all interview and translated into English; (2) familiarisation of the data by repeated reading of the transcripts; (3) generation of initial codes from the data inductively; (4) constructing an analytical framework using the COM‐B model, a deductive step where predefined theoretical constructs were used; (5) applying the COM‐B model systematically by X.Z., M.D., H.A.G. and A.F.; (6) charting data into the framework integrating inductive insights with the deductive structure of the COM‐B model; (7) data was grouped and main themes were identified, the data was interpreted and discuss with all authors.

### 2.6 Rigour and Trustworthiness

To ensure the rigour and trustworthiness of the findings, we adhered to four key criteria: transferability, dependability, confirmability and credibility (Guba and Lincoln 1985). Transferability was achieved through a thick description of the context, participants and methods, as well as purposive sampling and data saturation to guide data collection. To ensure dependability and confirmability, reflexive notes were kept by the first author during the study, demonstrating how interpretations were derived from the data. Additionally, rich quotes from the participants were provided to support the described themes and align the research team's interpretation with the data. Credibility was established through peer debriefing within the research team, which included expert qualitative researchers who reviewed the transcripts and discussed the research process. Furthermore, member checking was conducted through facilitated discussions with participants to review the research team's findings (Cypress 2017).

### 2.7 Ethical Consideration

Ethical approval was obtained from King's College London's ethics committee (LRS/DP‐22/23–36236) as part of the process for reviewing the study proposal by the ethics committee, participant recruitment and data collection were conducted online without hospital involvement. All participants provided informed consent. Participants were informed that they had the right to refuse to participate and were allowed to withdraw at any time during the study. Participant anonymity was protected, such that no personally identifiable information was attributed.

## 3 Findings

### 3.1 Characteristics of Participants

A total of 57 potential participants approached the research team, 34 did not participate for the following reasons: did not meet inclusion criteria (*n* = 27), no longer interested in participating (*n* = 3), lost to contact (*n* = 3) and inability to arrange interview due to a busy schedule (*n* = 1). Overall, 23 people with young‐onset type 2 diabetes from 13 different cities and 8 provinces in China participated in the interview. The participants were aged between 20 and 40 years with a median age of 29 years. Around half of the participants were female (*n* = 13). The median diabetes duration was 1 year (range 3 months to 11 years). Most participants were employed or self‐employed (*n* = 15), lived in an urban area (*n* = 19) and had no children living at home (*n* = 15). Most participants engaged in aerobic activity currently (*n* = 15). Full details of the participant characteristics and physical activity experience are presented in Tables 1 and 2.

**TABLE 1:** Participant characteristics (*N* = 23).

Variables	*n* (%)
Age, years	
Median (range)	29 (20–40)
IQR (lower quartile–upper quartile)	11 (23–34)
18–29	12 (52.2)
30–40	11 (47.8)
Gender	
Male	10 (43.5)
Female	13 (56.5)
Employment	
Employed or self‐employed	15 (65.2)
In education	4 (17.4)
No employment	4 (17.4)
Living	
Urban	19 (82.6)
Rural	4 (17.4)
Children in household	
Yes	8 (34.8)
No	15 (65.2)
Educational level	
Master/Bachelor	11 (47.8)
Junior college	7 (30.4)
High school/Junior high school	5 (21.7)
Diabetes complication	
Yes	3 (13.0)
No	19 (82.6)
Not sure	1 (0.4)
Family history	
Yes	12 (52.2)
No	10 (43.5)
Do not know	1 (0.4)
Diabetes duration, year (s)	
Median (range)	1 (0.25–11)
IQR (lower quartile–upper quartile)	1.67 (0.33–2)
< 1	8 (34.8)
1–5	11 (47.8)
> 5	4 (17.4)
Treatment regimen	
Oral medication alone	11 (47.8)
Insulin alone	2 (0.8)
No treatment or self‐care management alone	3 (13.0)
Oral medication & insulin	4 (17.4)
Oral medication & GLP‐1	3 (13.0)

*Note:* Values may not add up to 100% due to rounding.

Abbreviations: GLP‐1, glucagon‐like peptide‐1; IQR, interquartile range.

**TABLE 2:** Physical activity experience of participants (*N* = 23).

Variables	Current PA experience	PA experience before diabetes diagnosis
	*n* (%)	*n* (%)
Duration, minutes/week		
Median (range)	350 (50–700)	45 (0–420)
IQR (lower quartile–upper quartile)	320 (130–450)	180 (0–180)
Type		
Aerobic activity only	15 (65.2)	12 (52.2)
Resistance exercise only	1 (0.4)	/
Combined exercise	7 (30.4)	1 (0.4)
Almost no PA	/	10 (43.5)
Frequency, times/week		
< 3	4 (17.4)	15 (65.2)
3–5	11 (47.8)	4 (17.4)
> 5	8 (34.8)	3 (13.0)

*Note:* Values may not add up to 100% due to rounding.

Abbreviations: IQR, interquartile range; PA, physical activity.

### 3.2 Themes

**FIGURE 1:**
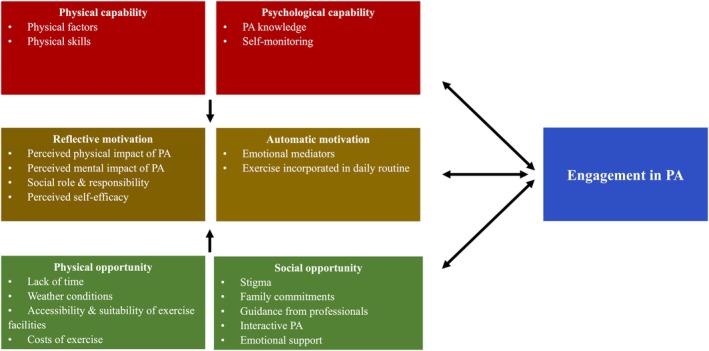
Summary and interaction of identified themes and subthemes mapped onto the capability, opportunity, motivation and behaviour model. PA, physical activity. [Colour figure can be viewed at wileyonlinelibrary.com]

The transcripts provided data that represent all components of the COM‐B model. Table 3 presents a summary of the COM‐B constructs, subthemes and supporting quotes. Six themes and nineteen subthemes were identified as barriers or facilitators to physical activity in people with young‐onset type 2 diabetes (Figure 1).

**TABLE 3:** Barriers and facilitators to physical activity categorised by COM‐B components.

COM‐B	Subthemes	Subtheme definition	Quotes
Capability			
Physical	Physical factors	Barriers: Physical health status (feel unwell, exercise type restriction due to overweight), tiredness or not enough rest	“If I have a cold, a fever, a headache, it affects my body function when I exercise. My body function reminds me that I need to rest. In this case, I may exercise less, well, the body has already sent a signal.” —S12 “If, for instance, you have poor sleep, you wouldn't want to engage in physical activities, you'd prefer rest and wouldn't want to engage in activities that consume physical energy.” —SP1
	Physical skills	Barriers: Lack of skills needed for PA	“I don't know how to swim. I know swimming is good, as even the doctor recommended it, but I can't.” —S21
Psychological	PA knowledge	Barriers: Misperception of physical activity, for example, perceived conflict between muscle growth and diabetes management Facilitators: Knowledge of PA (knowledge includes PA definition, benefits and common PA types; increased knowledge through APP or web‐based information)	“You know, gaining muscle and losing weight are conflicting. Oh, there's a problem—there's a problem because you need to control blood sugar, so you can't eat too much, and the calorie intake needs to be controlled. However, when you exercise, if you want to build muscle, you have to eat more. Actually, at the moment, this is quite contradictory for me.” —S2 “My definition of exercise is involving overall body movement. For example, walking or brisk walking all count as exercise, and for a period, I also did some aerobic exercises like the ones by Pamela (fitness influencer), where I sweat a lot.” —S7
	Self‐monitoring	Facilitators: Monitoring calorie consumption, heart rate, step, exercise time and speed through activity tracker; recording step and exercise time through fitness Apps	“It's a Xiaomi band, mainly for checking my steps and sometimes my heart rate.” —S20
Opportunity			
Physical	Lack of time	Barriers: Work overtime, workload of job, sedentary job, family commitment, at the stage of transitioning from a student to a professional	“I have an internship, after work, I get back around 5:30 pm, have dinner, then take a shower, because I usually wake up around 7 am every morning, I sleep early, and there's no extra time for exercise.” —S19
	Weather conditions	Barriers: Hot, rain, cold weather	“The main reason hindering my exercise is the heat. It's too hot at present, and it's a bit of a barrier.” —SP1
	Accessibility & suitability of exercise facilities	Barriers: Lack of exercise facilities such as professional running track, exercise place; cleaning issue using outside facilities; privacy and safety problem in exercise place Facilitators: Access to exercise facilities (exercise equipment support from family, company and community; exercise site support from company, community and college)	“The government promotes exercise, but my residential area doesn't have running tracks or any places for exercise.” —S7 “There is fitness equipment that is a bit like that in the park, and then there is the court for playing badminton, and then you can play volleyball, and there is fitness track.” —S3
	Costs of exercise	Barriers: Financial expense of exercise Facilitators: Financial support from family; economic incentives and exercise expense subsidies from company	“Currently, playing table tennis is very costly. It's very expensive. A bucket of balls costs over 70 yuan for 12 balls, and it's gone after playing three times. Each time, the venue fee is 50 yuan. Just thinking about it, it's already very expensive.” —SP1 “Our company also allocates funds for physical activities, which is several thousand yuan a year per person… this is equivalent to giving you a subsidy, and then you can sign up to go to a gym.” —S1
Social	Stigma	Barriers: Negative words from colleagues, friends and family; negative attitude from boss	“It's like, um, every time when I do some sports, for example, after my workout, I take some screenshots of how much I ran or what I did for exercise today, then they see it, and they just think it's unnecessary. Some say you'll give up after 2 or 3 days, different phrases like that.” —S19
	Family commitments	Barriers: Take care of child, upset family members due to not enough company time Facilitators: Childcare and housework as a type of physical activity	“So, after dinner, I really wish to go for a walk, but there are tasks like the child's homework, and I just don't have the time for exercise.” —S21
	Guidance from professionals	Barriers: Lack of guidance or consultation from professionals (e.g., lack of platform with a mix of professionals including HCP, nutritionist and professional trainer) Facilitators: Health education/advice by HCP, individualised exercise plan	“I would like to exercise, but I don't know how to exercise professionally, because I have told you before that I bought a fitness card, and I have been to that gym, but the gym coach there may be professional in sports and yoga, but he doesn't know much about diabetes, so he couldn't give me a reasonable exercise suggestion.” —S21
	Interactive PA	Barrier: Isolation or alone Facilitators: Exercise group or club (company badminton club, company fitness club, general exercise group); organised sports activities (college, company, government); exercise buddy (family, friends, diabetes peers or others); competitive PA (badminton competition and step competition by company, updated steps in social media)	“I've considered cycling, but I feel a bit unsuitable doing these activities alone.” —S18 “… Someone to organise, like group‐oriented exercise. Organised exercises are better to mobilise both group and personal positivity.” —S16 “After exercising every day, isn't it that WeChat sports are updated at night, and then I see that my step count is much higher than many friends, and then I am happier. And there will also be friends who come to ask, what are you doing every day, how do you walk so many steps, I will tell them that I am exercising every day, and it is more joyful.” —S4
	Emotional support	Barriers: Lack of emotional support or empathy during consultation with HCP Facilitators: Peer emotional support from other people with diabetes or diabetes online groups; encouragement from family and friends; exercise reminding by HCP, family, friends through phone call or text message	“When I was overweight, they encouraged me to exercise. My close friends said, ‘After exercising, you'll have a better body shape, and you'll become more confident.’ They encourage me to keep going and do my best.” —S13 “I feel people with diabetes, especially younger ones, face a big emotional blow after being diagnosed. I don't know about others, but in my memory, I always thought only doctors had that ability. When I found out I had the disease, I desperately hoped doctors would pay more attention to me. I trusted what they said 100%, but they didn't. I remember very clearly that the doctor who diagnosed me said it was nothing to worry about and just told me to go back and treat it or do what I needed to do.” —S10
Motivation			
Reflective	Perceived physical impact of PA	Barriers: Physical change after exercise (blood sugar rises after exercise, blood sugar fluctuation, early‐stage weight regain, chubby now‐won't look good if increase muscle), PA has limit impact on diabetes control Facilitators: Better physical health (accelerate metabolism, loss weight, avoid muscle atrophy, avoid or control complication, boost immunity, glycaemic control, improve insulin sensitivity, improve sleep quality, reduce body fat, improve physical fitness, physical function recovery), better appearance (better body shape, build muscle, getting leaner, better skin, body age younger than actual age), more energetic	“Because walking doesn't have a noticeable effect on my blood sugar, I'm lazy to move; it's not motivating for me. Even walking for an hour or an hour and a half doesn't lower my blood sugar, so I'm lazy to exercise, no motivation for me…After meals, it's around 8 or 9, when you return from exercise, if you stop, it rises. It's tough” —S20 “Apart from lowering blood sugar, it's good for your health and boosts your immunity.” —S14 “I don't think about anything else, just lifting dumbbells. Then I think, wow, my body shape becomes perfect, with an eight‐pack. Wow, the body looks great, and then I'm motivated to exercise again.” —S11 “What does exercise mean to me? It can allow me to stop taking medication.” —S6
	Perceived mental impact of PA	Facilitators: Better mental health (feel more fulfilled, reduce negative emotions, reduce overthinking, reduce stress, less worried, relaxed, improved self‐confidence, good mood, happier through increased dopamine)	“It might increase my focus on exercise, reducing the time for other thoughts like salary increases, future, or worrying about others' opinions. I don't have time to think about this, so I focus on exercise, which will reduce anxiety or various negative emotions.” —S12
	Social role & responsibility	Barriers: De‐prioritising self over others, for example, work, family; gender (being female) Facilitators: Responsibility for dog, happy family; saving lives in critical moments if acquire other PA skills; physically active professionals	“If I allocate more time for exercise, it becomes challenging to spend time with my family and do other things.” —SSI2 “Interviewer: What motivates or could motivate you to be physically active? SSI18: Because of my dog. Well, because my dog doesn't relieve himself inside the house, he needs to go outside daily. So, I walk the dog every day because of this.” —S18
	Perceived self‐efficacy	Facilitators: Self‐discipline/self‐belief, self‐awareness of more exercise Barriers: Negative self‐identify (laziness)	“At least now, I'm aware of the need for physical activity. Previously, I was negligent and didn't care, but after diabetes, I find it important.” —SP2 “For me, I'm lazy when it comes to exercise.” —S20
Automatic	Emotional mediators	Barriers: General negative emotion could lead to low motivation for exercise; perceived anxiety from exercise content in social media; worried about privacy and safety problem in exercise place; a sense of boredom from the repetitious nature of exercise; fear of getting injured; afraid of judgement; disappointment in lack of glycaemia improvement through exercise Facilitators: Enjoyment or fun in exercise, passion in exercise, fear of diabetes complication, fear of death	“When I argue with my husband… when we give each other the silent treatment, it makes me emotionally negative, and those days I don't feel like exercising, I just want to go home and sleep.” —S13 “It becomes very boring. Sometimes I feel it's so boring. I lift dumbbells every day, and it's always the same few movements. I find it very boring and a little hard to keep going.” —S11 “It's more of a sensation near the ankle joint during the start of running… I am worried about the risk of a leg injury, but other than that, everything is fine. This is my main concern.” —S19
	Exercise incorporated in daily routine	Facilitators: Exercise has been a part of daily life, housework‐related PA (e.g., childcare), work‐related PA	“If I skip exercising for a few days, I feel uncomfortable and itchy, I want to exercise, it's become a habit, and even if I'm lazy for a few days, I feel bad if I skip exercising for too long.” —S13 “… It will also affect it. For me, they may be regarded as a kind of exercise. For example, I need to take care of the child, to hold the child, and then take him out to play. In fact, this is a kind of exercise for me, because in the process of chasing him, I am also running and consuming (energy).” —S3

Abbreviations: COM‐B, capability, opportunity, motivation and behaviour; HCP, health care professional; PA, physical activity; S No. , semi‐structured interview No. ; SP No., semi‐structured pilot interview No.

#### 3.2.1 Physical Capability

##### 3.2.1.1 Physical Factors

Some participants reported how various physical issues such as fatigue, being overweight, feeling unwell created barriers to engaging in physical activity. Participants with fatigue often prioritised resting rather than exercising in their free time, as they felt that physical activity consumes too much physical energy. Two individuals who were overweight thought that their physical condition restricted the type of exercise they could do and that they felt physically uncomfortable when performing exercises like jumping or running. They were also concerned that resistance exercise might increase their physical size further. Additionally, people reported that they often felt unwell, and this reduced their ability to engage in physical exercise.

If, for instance, you have poor sleep, you wouldn't want to engage in physical activities, you'd prefer rest and wouldn't want to engage in activities that consume physical energy. SP1

##### 3.2.1.2 Physical Skills

Most participants reported that they were currently involved in physical activities such as walking, running, cycling, badminton and dumbbell lifting. However, other physical activities that required a specific skill were viewed as being more challenging, such as swimming.

I don't know how to swim. I know swimming is good, as even the doctor recommended it, but I can't. S21

#### 3.2.2 Psychological Capability

##### 3.2.2.1 Physical Activity Knowledge

Most participants had a good understanding of physical activity and could identify the benefits of physical activity and exercise types.

My definition of exercise is involving overall body movement. For example, walking or brisk walking all count as exercise, and for a period, I also did some aerobic exercises like the ones by Pamela (fitness influencer), where I sweat a lot. S7

Some participants reported that their knowledge was enhanced through online website searching, fitness Apps and online diabetes groups. However, other participants reported that they did not understand how exercise might benefit their diabetes or that it may contradict their goal to lose weight.

##### 3.2.2.2 Self‐Monitoring

Some participants used self‐monitoring technology such as fitness apps, to monitor physical activity, via wearable devices that: tracked physical activity (daily steps, exercise time and exercise speed); monitored their heart rate and calorie consumption.

#### 3.2.3 Physical Opportunity

##### 3.2.3.1 Lack of Time

More than half of participants reported insufficient time as a barrier to physical activity, primarily due to work and family responsibilities. Family commitment, such as childcare, accompanying family members, and looking after elders, constrained their exercise time. Additionally, factors like heavy workload, overtime, sedentary work and the transition from a student to professional are also common barriers to physical activity in Chinese people with young‐onset type 2 diabetes. One participant, a programmer, mentioned having to work on *‘bugs’* outside of official work hours, leading to work‐life imbalance and less engagement in physical activity.

##### 3.2.3.2 Weather Conditions

Some participants reported that weather conditions impacted on their physical activity engagement, they would be less inclined to exercise outdoors if it was too hot, cold or rainy. For people who exercised in the gym or at home, the weather was not reported as a hinderance for physical activity.

The main reason hindering my exercise is the heat. It's too hot at present, and it's a bit of a barrier. SP1

##### 3.2.3.3 Accessibility & Suitability of Exercise Facilities

The availability of exercise facilities in the work place, community or college made physical activity easier. However, some individuals expressed dissatisfaction with the level of professionalism of the exercise facilities (e.g., absence of professional running track) or limited exercise space. One participant reported that hygiene was a barrier to using outside public exercise equipment, while two participants reported safety and privacy issues in outdoor exercise places.

##### 3.2.3.4 Costs of Exercise

Some participants reported that the financial expense of exercise equipment or exercise venue was a barrier to exercise:

Currently, playing table tennis is very costly. It's very expensive. A bucket of balls costs over 70 yuan for 12 balls, and it's gone after playing three times. Each time, the venue fee is 50 yuan. Just thinking about it, it's already very expensive. SP1

A few participants indicated that financial support from families facilitated them to engage in physical activity. One participant received exercise expense subsidies from his work place, which also offered economic incentives to the team that accumulated the most steps in a step competition. The participant considered this incentivisation approach to be an effective means of promoting physical activity, serving as a strong motivator for initiating exercise and playing a crucial role in sustaining consistent engagement in physical activity.

#### 3.2.4 Social Opportunity

##### 3.2.4.1 Stigma

The negative societal perception toward individuals who are overweight or have diabetes was identified as a significant barrier to physical activity. Some participants reported experiencing stigma in social‐based physical activity environment.

It's like, um, every time when I do some sports, for example, after my workout, I take some screenshots of how much I ran or what I did for exercise today, then they see it, and they just think it's unnecessary. Some say you'll give up after 2 or 3 days, different phrases like that. SP19

The attitude of line managers toward employees or postgraduate students was reported as a crucial factor for the engagement in physical activity in this study. For example, one participant mentioned that her boss was reluctant about her regular exercise routine because it resulted in fewer hours of overtime, which was seen as reducing her availability and commitment at work.

##### 3.2.4.2 Family Commitments

Some participants reported that family commitments were a barrier to physical activity. Childcare was particularly an issue for all the mothers who were interviewed. Their priorities were to ensure that care was provided for their babies or assist their children with schoolwork. Family commitments in this study also included spending time to accompany family and taking care of elders.

So, after dinner, I really wish to go for a walk, but there are tasks like the child's homework, and I just don't have the time for exercise. S21

However, two participants believed that housework and childcare could be viewed as a type of physical activity. They believe that these activities helped them expend energy.

##### 3.2.4.3 Guidance From Professionals

Most participants reported that the professional guidance can be a barrier or facilitator to physical activity. A few individuals reported that health education on diabetes or physical activity provided by health care providers had increased their knowledge, leading to improved engagement in physical activity. Two participants mentioned that an individualised exercise plan by a personal trainer or physiotherapist had helped or would help them improve their participation in physical activity. However, half of the participants reported that the absence of guidance or consultation from professionals (e.g., health care professionals, nutritionist, trainer, physiotherapist) in their daily life can be a crucial barrier to physical activity engagement and diabetes management.

I would like to exercise, but I don't know how to exercise professionally, because I have told you before that I bought a fitness card, and I have been to that gym, but the gym coach there may be professional in sports and yoga, but he doesn't know much about diabetes, so he couldn't give me a reasonable exercise suggestion. S21

##### 3.2.4.4 Interactive Physical Activity

The context in which physical activities takes place can be individual or social. Most participants reported that interactive physical activity with others helped their engagement in physical activity. The participants identified a range of interactive exercise contexts: exercise groups or clubs (badminton club and fitness club organised by company, general exercise group); organised sports activities by college, company and government; exercise buddies (e.g., family, friends); competitive physical activities (badminton and step competition organised by company, updated steps in social media). Conversely, exercise alone without interactive components was reported by some participants as a barrier to physical activity and lowered their motivation to exercise.

I've considered cycling, but I feel a bit unsuitable doing these activities alone. S18

##### 3.2.4.5 Emotional Support

The key sources of emotional support (e.g., encouragement) for exercise identified by participants were from family, friends, peers with diabetes or diabetes online groups. Some participants also reported that exercise reminder calls or text messages aided in enhancing their physical activity level, and regular follow‐up calls by health care professionals would help people who *were not disciplined and indulgent themselves* engage in more exercise. Additionally, it is important to note that some participants reported experiencing a lack of empathy from health care professionals during consultation; especially for young people who were newly diagnosed with diabetes. They felt a *big emotional blow* and expressed a desire for health care professionals to provide greater support and attention.

#### 3.2.5 Reflective Motivation

##### 3.2.5.1 Perceived Physical Impact of Physical Activity

Almost all participants reported that their perception of the physical impact of physical activity either hindered or facilitated their engagement in physical activity. Most participants believed that there were numerous beneficial impacts from physical activity, including better physical health (glycaemic control, weight loss, avoid muscle atrophy, boost immunity, improve insulin sensitivity, improve sleep quality, reduce body fat, improve physical fitness), as well as better appearance (better body shape, build muscle, getting leaner, better skin, body's age younger than actual age). However, some people had negative perceptions about the experience of exercise, such as it increased their glucose levels, or altered their body shape by increasing muscle mass, which had a negative impact on their body image. Additionally, some people perceived limited benefit in glycaemic control from physical activity.

Because walking doesn't have a noticeable effect on my blood sugar, I'm lazy to move; it's not motivating for me. Even walking for an hour or an hour and a half doesn't lower my blood sugar, so I'm lazy to exercise, no motivation for me…After meals, it's around 8 or 9, when you return from exercise, if you stop, it rises. It's tough. S20

##### 3.2.5.2 Perceived Mental Impact of Physical Activity

Many participants reported mental health benefits from physical activity and that this motivated their physical activity. They believed that physical activity could boost their self‐confidence, enhance their mood and provide a sense of fulfilment. Additionally, negative emotions such as worrying, stress, anxiety and overthinking were reduced through physical activity.

It might increase my focus on exercise, reducing the time for other thoughts like salary increases, future, or worrying about others' opinions. I don't have time to think about this, so I focus on exercise, which will reduce anxiety or various negative emotions. S12

##### 3.2.5.3 Social Role & Responsibility

Social roles and responsibilities of young adults with type 2 diabetes were reported as mediators of physical activity. Young adults hold multiple roles, such as being employers or employees, wives or husband, sons or daughters, pet raisers, mothers or fathers. Most participants believed that these social roles were barriers to physical activity. The male participants tended to prioritise work over their personal needs. Female participants often gave precedence to family duties, such as childcare over their own demands. Family was also reported as a facilitator for women since the desire for a happy family prompts them to engaged in physical activity to minimise the negative influence of diabetes. Some female participants reported their gender was a cause of safety and privacy concerns that resulted in a barrier to physical activity. They expressed concerns about sexual harassment and other forms of violence in public space, which they believed are common occurrences for women worldwide and that public facilities were more frequently used by men instead by women. Additionally, physically activity incorporated into a daily routine made exercise easier, such as physically active work or being a pet owner.

…Because of my dog. Well, because my dog doesn't relieve himself inside the house, he needs to go outside daily. So, I walk the dog every day because of this. S18

##### 3.2.5.4 Perceived Self‐Efficacy

Some participants reported negative self‐identify (being labelled as lazy) as a barrier to physical activity. They lacked confidence in their ability to maintain regular physical activity. Though some of them were aware of the potential complications of diabetes, they did not believe that these diabetes complications would manifest at a young age. On the contrary, some other participants believed that the key determinant to physical activity is self‐discipline or self‐belief. They firmly believed in their capacity to maintain exercise, which they saw as a vital tool in managing their diabetes.

#### 3.2.6 Automatic Motivation

##### 3.2.6.1 Emotional Mediators

Emotional factors were frequently reported by participants as facilitators or barriers to engagement in physical activity. Some physically active interviewees expressed a sense of fun and enjoyment while exercising, particularly when they were passionate about a particular exercise or a sport where they noticed physical improvements in their skill. Others were driven to exercise for less positive reasons, due to fear of future diabetes complications or early death. Negative emotions acted as barriers to physical activity for some participants, manifesting in various ways. Low mood and general negative emotion diminished their motivation to engage in exercise. Anxiety stemming from misleading exercise content on social media—such as exaggerated claims about topical fat loss or the severity of their condition—contributed to uncertainty. Concerns about privacy and safety in exercise environments created hesitation. Fear of injury and apprehension about being judged by others also caused avoidance behaviours. Some participants experienced disappointment when their efforts did not lead to noticeable improvements in glycaemic control. Additionally, the repetitive nature of certain exercises led to boredom, making sustained engagement difficult.

It becomes very boring. Sometimes I feel it's so boring. I lift dumbbells every day, and it's always the same few movements. I find it very boring and a little hard to keep going. S11

##### 3.2.6.2 Exercise Incorporated in Daily Routine

Some physically active participants reported that their current high physical activity levels were facilitated by their daily routines (e.g., housework) which increased their daily step count. Additionally, some participants reported that structured exercise training had become a habit and was seamlessly incorporated into their daily lives.

If I skip exercising for a few days, I feel uncomfortable and itchy, I want to exercise, it's become a habit, and even if I'm lazy for a few days, I feel bad if I skip exercising for too long. S13

## 4 Discussion

This is the first qualitative study to advance knowledge of the barriers and facilitators to physical activity in individuals with young‐onset type 2 diabetes. The findings demonstrated a comprehensive perception from participants regarding a variety of barriers and facilitators to physical activity across all domains of the COM‐B model. Particularly the domains of psychological capability, social opportunity and reflective motivation emerge as crucial areas for developing strategies to enhance physical activity levels in this demographic. The interplay between various COM‐B model domains highlights the multifaceted nature of physical activity engagement.

Psychological capability, which includes knowledge, mental skills and self‐regulation, refers to the ability to engage in the necessary cognitive processes to perform a behaviour (Bandura 2001; Michie et al. 2011). In this study, the subthemes of physical activity knowledge and self‐monitoring emerged in the domain of psychological capability. Most participants demonstrated a good understanding of physical activity, and all domains of physical activity (domestic, transportation, occupational and leisure time physical activity) were covered in the findings. However, misperceptions persisted such as equating physical activity solely with planned, structured exercise and perceiving a conflict between diabetes management and muscle building. Our findings are consistent with prior research emphasising the critical role of knowledge in being psychologically capable to perform physical activity in young adults (Peng et al. 2023; Wong et al. 2023). This underscores the importance of tailored education and skill development programmes to enhance the engagement in physical activity. The study also highlights the role of self‐monitoring of physical activity (e.g., heart rate, step, calorie, exercise time and exercise speed) is a facilitator to increase physical activity. Our finding supported evidence (Ferguson et al. 2022; Rossen et al. 2021) that self‐monitoring serves as a powerful behaviour change technique to substantially decrease sedentary behaviour and increase physical activity. Findings from this study also support the theoretical domains framework, which suggests self‐regulatory processes, such as self‐monitoring, are necessary for developing the psychological capacity to engage in a behaviour (Cane et al. 2012). Furthermore, our findings highlighted the vital role of technology in promoting physical activity. People reported self‐monitoring of physical activity with usage of fitness Apps and activity trackers, increased physical activity knowledge in daily life with fitness Apps and online forums, indicating a profound impact on physical activity with technological advance.

The influence of health promotion and public health fields has led to a greater emphasis on the availability and accessibility, or environmental opportunities, both social and physical. Social opportunity afforded by the cultural milieu dictates the way that people perceive and approach things (Michie et al. 2011). The findings of this study suggest that stigma due to diabetes or overweight were barriers to physical activity in individuals with young‐onset type 2 diabetes. Stigma is characterised by labelling, stereotyping, separation, status loss and discrimination in power situation, and often fuelled by false ideas about the causes of health conditions (e.g., diabetes, obesity) (Westbury et al. 2023). Our study supported this point, with some participants being criticised or blamed by their family members due to individual factors (i.e., they would not have diabetes or obesity if they exercised more, had less unhealthy food and drinks), without considering genetic and environmental factors. Stigma was reported to be largely related to younger adults with diabetes (Eitel et al. 2023; Puhl et al. 2020), exposure to stigma in people with diabetes can cause depression, lower self‐esteem, social comparisons, worse self‐care behaviours (e.g., physical inactivity), ultimately result in various health complications and premature mortality. These highlighted the importance of mental health and emotional support from society for physical activity in people with young‐onset type 2 diabetes. Findings from this study also support that social influences or interpersonal processes can lead individuals to change their feelings, thoughts or behaviours (Cane et al. 2012). For example, Encouragement and exercise reminder from family, friends, diabetes peers and health care providers was a vital facilitator to physical activity, which is also consistent with prior studies (Peng et al. 2023; Wong et al. 2023). Some participants reported experiencing lack of empathy from health care providers. As low emotional support has been associated with increased diabetes distress and heightened self‐stigma among people experiencing poor interactions with health care professionals (Celik et al. 2023; Puhl et al. 2020), we suggested emotional support by health care providers during consultations to improve self‐care engagement and diabetes outcomes.

Motivation comprises internal organismic processes that influence behaviour, reflective aspect involves conscious and purposeful consideration (Michie et al. 2011; Rosenkranz et al. 2023). Perceived self‐efficacy, that is, an individual's belief in their own capability, is a key subtheme of reflective motivation and essential psychosocial determinant of behaviour (Wong et al. 2023) and was found to help to sustain physical activity levels in people with young‐onset type 2 diabetes in this study. This observation is consistent with prior research indicating that perceived competence managing diabetes‐related behaviours is associated with improved self‐care practices (such as regular physical activity) (Zhao et al. 2023). Conversely, participants who were less active frequently cited negative self‐beliefs, such as perceptions of laziness. Similarly, this study showed that individual perceptions of outcomes (physical and mental impact of physical activity) could be a vital factor in health behaviour. This finding suggests a potential age‐related difference in individual perceptions, as compared to previous evidence (Bethancourt et al. 2014; Maula et al. 2019; Peng et al. 2023; Wong et al. 2023). While health outcomes remain a primary motivator factor for physical activity across all ages, for middle‐age and older adults, health is the most cited and prioritised facilitator to physical activity (Bethancourt et al. 2014; Maula et al. 2019). In contrast, younger individuals with type 2 diabetes often prioritise appearance‐related outcomes (such as improved body shape, better skin, younger biological age than chronological age), along with psychosocial benefits like enhanced self‐confidence (Peng et al. 2023; Wong et al. 2023). These differences can be attributed to the nuanced interplay of social and cultural contexts in young‐onset type 2 diabetes (Peng et al. 2023; Wong et al. 2023).

Social roles and responsibilities were identified as key factors in the reflective motivation for physical activity, which indicated an epitome of traditional patriarchal views. These cultural norms, which typically see the men as breadwinners and women as homemaker, have been linked to have adverse health outcomes in evolutionary anthropology studies (Sear 2021), still manifests in our study. Lack of time for physical activity due to increased working hours was reported by males, and lack of time due to family commitment was more often reported by females. This issue is particularly pronounced among young adults who often juggle multiple roles and responsibilities such as employees, parent, partner, caregiver and pet raisers, leading to motivation to exercise. Additionally, women in this study expressed concerns over privacy and safety issues, which deterred them from participating in public exercise facilities and gyms. This pattern of socio‐cultural and environmental barriers affecting women's physical activity is consistent with existing evidence not only in Asia but in western countries and around the world (Laar et al. 2022; Sear 2021; Wong et al. 2023). To address these disparities, a sustained effort from individuals, communities, organisations and policymakers is needed to break gender stereotypes and improve physical activity level in young adults with type 2 diabetes (Peng et al. 2023).

The barriers and facilitators to physical activity in individuals with young‐onset type 2 diabetes are diverse at different domains in COM‐B model and intertwined. People without specific physical skills (physical capability) might be afraid of injury (automatic motivation) during exercise. People tend to have belief in ability and outcomes of physical activity (reflective motivation) with required physical skill, good physical condition (physical capability) and sufficient knowledge (psychological capability). Self‐monitoring of physical activity (psychological capability) facilitates incorporation of exercise in daily routine (automatic motivation). Furthermore, people tend to prioritise work and family (physical and social opportunity) over their own needs due to their social roles and responsibilities (reflective motivation). People engaging in interactive physical activity (social opportunity) tend to have more perceived enjoyment and passion (automatic motivation). Stigma (social opportunity) relates to lower self‐efficacy and higher diabetes distress (reflective and automatic motivation). Addressing these complex and interconnected factors through tailored interventions could significantly enhance physical activity engagement among this population.

### 4.1 Implications

Based on the findings from this qualitative study, physical activity in individuals with young‐onset type 2 diabetes can be enhanced through multilevel strategies focusing on psychological capability, social opportunity and reflective motivation. The findings from this study indicate a need for a multilevel approach to enhancing physical activity that includes doctors, diabetes specialised nurses and professional trainers, in the form of supportive group, forming an environment that supports people with young‐onset type 2 diabetes. Accessible and affordable technology (e.g., fitness Apps, wearable activity tracker, virtual reality fitness) is an ideal context in which to improve physical activity levels in young adults with type 2 diabetes. Local authority or government should make professional exercise facilities accessible and free to public especially in remote areas. The findings showed the importance of interactive physical activity in people's daily life for maintaining and promoting physical activity and is suggested to be involved in intervention design for increase physical activity. Tailored physical activity is recommended since some barriers and facilitators are on an individual level (e.g., rising blood sugar following exercise, flat foot, different exercise level). Diabetes knowledge should be available to general public especially the causes of the condition by government and health care system to reduce stigma. Actions should be in place to break gender stereotype and promote gender equality for health outcomes and well‐being. By taking these steps, a holistic and multilevel strategy involving psychological capability, social opportunity and reflective motivation should be promoted for increasing physical activity level in people with young‐onset type 2 diabetes.

### 4.2 Strengths and Limitations

This is the first qualitative study to explore the barriers and facilitators to physical activity among people with young‐onset type 2 diabetes using the COM‐B model. This study provided insight to help inform the development of future tailored interventions to improve physical activity in young adults with type 2 diabetes.

However, there were some limitations in this study. The authors could not precisely identify the intensity of physical activity of participants, which was mostly vaguely described during the interviews, either due to subjective interpretations of intensity or engagement in a mix of activities that differ significantly in intensity. This can be addressed by asking for objective measures if possible or using a mixed methods approach in future studies to offer precision and objectivity. The findings from the purposive sampling technique may lack transferability to the larger population (Moser and Korstjens 2018). Additionally, most participants were well‐educated, living in urban areas, though theses demographics appear to be common in young adults, the findings should be interpreted with caution since theses demographics tend to be associated with higher levels of physical activity.

## 5 Conclusion

We have identified a comprehensive set of barriers and facilitators that merit consideration in promoting physical activity in young‐onset type 2 diabetes using the COM‐B model as a framework. We observed interactions between different levels of domains in COM‐B model. The findings suggest that increasing knowledge, leveraging technology for self‐monitoring and combating societal stigma are crucial steps toward promoting a more active lifestyle in this population. Additionally, the study highlights the need for supportive environments that consider individual self‐efficacy and motivations, including health, aesthetics and psychosocial well‐being. Collaborative efforts are needed to create accessible, inclusive environments that encourage physical activity. Tailored intervention strategies, emphasising emotional support and acknowledging the complex roles and responsibilities of young adults, are essential for improving physical activity levels in this population.

## Author Contributions

X.Z. was involved in conceptualization, data curation, interviews, formal analysis, methodology, project administration, writing – original draft. M.D. and H.A.G. were involved in conceptualization, data curation, formal analysis, methodology, supervision, writing – review and editing. X.G. was involved in formal analysis, writing – review and editing. A.F. was involved in conceptualization, data curation, formal analysis, methodology, funding acquisition, supervision, writing – review and editing. All authors approved the final draft of the manuscript.

## Conflicts of Interest

The authors declare no conflicts of interest.

## Supporting information


Appendix S1.

Data S1.

## Data Availability

The data supporting the findings of this study are available from the corresponding author upon reasonable request. The data are not publicly available due to privacy or ethical restrictions.
