# Supplementary material for: Barriers and Facilitators to Physical Activity in People With Young‐Onset (Aged 18‐40 Years) Type 2 Diabetes: A Qualitative Study
Source: J Clin Nurs. 2025 Feb 18;34(6):2386–99. doi: 10.1111/jocn.17691 (PMC12125533; doi:10.1111/jocn.17691)
Supplement: Supplementary file 1 — Appendix S1. [file JOCN-34-2386-s001.docx]

**Appendix 1 Participant recruitment advertisement**

**Appendix 2 Interview topic guide**

**Introduction and icebreaker questions**

--Introduce myself and thank the participants for agreeing to take part in the interview.

--Have you participated in a research interview before?

--The interview will focus on the experiences of physical activity before and after being diagnosed with T2D, as well as exploring the barriers and facilitators to physical activity.

--How long the interview will take

-- Some of the things I ask might seem obvious, but I want to know about what you think, and about your understanding, so I will ask you to explain things in your words.

--Do you have any questions?

**Demographic and clinical questions**

-- How old are you? What is your Gender?

-- What is you occupation/ level of education?

-- How would you describe your ethnicity?

--Do you have any religion believe?

--Do you have any diabetes complication? _____________

-- What’s your treatment regimen?

-- Do you have family history?

**Capability**

**How do you define the term “physical activity”?**

--Can you give me an example of a physical active?

--What do you think are the benefits of physical activity?

o How do you think being active would help you?

**Experience**

**Can you tell me about your physical activity experience before and after being diagnosed with T2D?**

--Frequency, intensity, duration of physical activity

--What physical activities do you like? (walking, running, swimming, etc.)

--Has it changed /how after being diagnosed with T2D?

**How easy/ difficult would be to increase your levels of physical activity?**

**Opportunity**

**What do you think are the main challenges to participate in physical activity?**

--Are there particular individuals/groups that would discourage of you being physically active?

--Think about the environment around you/ local facilities, and your local neighbourhood, how does this make it difficult to be active?

o How did you handle these difficulties? / How do you think you can tackle these barriers to physical activity?

**What do you think makes (would make) it easier for you to take part in physical activity?**

-- Are there individuals/groups that would support/ encourage you to be physically active?

o Emotional/practical support/ technology (e.g., text message, activity tracker, web-based information), explore in detail and related experience.

o If no response: for example, is there anyone that would offer to join in with you, or look after your child, or offer you a lot of encouragement?

--Think about the environment around you/ local facilities, and your local neighbourhood, how does this support you to be active?

**Motivation**

**What motivates/could motivate you to be physically active?**

--What does physical activity mean to you?

--How do you feel about your current levels of physical activity?

--How would you expect to feel if you increase or maintain them? What difference would it make to your life?

--What advice would you give to the people with young-onset T2D to improve their physical activity level?

**Closing question**

Is there anything else that you would like to add?
